# Unexplored Arsenals of Legume Peptides With Potential for Their Applications in Medicine and Agriculture

**DOI:** 10.3389/fmicb.2020.01307

**Published:** 2020-06-18

**Authors:** Rui M. Lima, Salome Kylarová, Peter Mergaert, Éva Kondorosi

**Affiliations:** ^1^Institute of Plant Biology, Biological Research Centre, Szeged, Hungary; ^2^Université Paris-Saclay, CEA, CNRS, Institute for Integrative Biology of the Cell (I2BC), Gif-sur-Yvette, France

**Keywords:** *Medicago truncatula*, nodule-specific cysteine-rich peptide (NCR), antimicrobial peptide (AMP), antibacterial activity, antifungal activity, ESKAPE bacteria, multifunctional roles

## Abstract

During endosymbiosis, bacteria live intracellularly in the symbiotic organ of their host. The host controls the proliferation of endosymbionts and prevents their spread to other tissues and organs. In Rhizobium-legume symbiosis the major host effectors are secreted nodule-specific cysteine-rich (NCR) peptides, produced exclusively in the symbiotic cells. NCRs have evolved in the Inverted Repeat Lacking Clade (IRLC) of the *Leguminosae* family. They are secreted peptides that mediate terminal differentiation of the endosymbionts, forming polyploid, non-cultivable cells with increased membrane permeability. NCRs form an extremely large family of peptides, which have four or six conserved cysteines but otherwise highly diverse amino acid sequences, resulting in a wide variety of anionic, neutral and cationic peptides. *In vitro*, many synthetic NCRs have strong antimicrobial activities against both Gram-negative and Gram-positive bacteria, including the ESKAPE strains and pathogenic fungi. The spectra and minimal bactericidal and anti-fungal concentrations of NCRs differ, indicating that, in addition to their charge, the amino acid composition and sequence also play important roles in their antimicrobial activity. NCRs attack the bacteria and fungi at the cell envelope and membrane as well as intracellularly, forming interactions with multiple essential cellular machineries. NCR-like peptides with similar symbiotic functions as the NCRs also exist in other branches of the *Leguminosae* family. Thus, legumes provide countless and so far unexplored sources of symbiotic peptides representing an enormous resource of pharmacologically interesting molecules.

## Introduction

Legumes are particular because they can form symbiosis with nitrogen fixing bacteria, which convert the atmospheric nitrogen into ammonia and satisfy the nitrogen need of the host plant (Graham and Vance, 2003). The symbiotic rhizobium partners are soil-dwelling alpha- or beta-proteobacteria, which are present intracellularly in the symbiotic organ, the root nodule, and are called bacteroids. The bacteroid-containing nodule cells become polyploid, grow to an extreme size, and host thousands of bacteroids (Kondorosi et al., 2013). In many legumes, the nitrogen fixing bacteroids are similar to cultured bacteria, which can change their lifestyle reversibly between the free-living and symbiotic states. In IRLC legumes or in certain legumes from the Dalbergioid clade, the bacteroids undergo an irreversible, terminal differentiation. This terminal differentiation is associated with definitive loss of cell division potential, changes in the membrane composition and permeability, cell growth from moderate to extreme sizes coupled to genome amplification, altered cell morphology (Mergaert et al., 2006; Montiel et al., 2017), and more efficient nitrogen fixation (Oono and Denison, 2010). To accomplish this, legumes have evolved a spectacular arsenal of antimicrobial peptides (AMPs) which are targeted to the bacteroids and provoke their differentiation (Mergaert, 2018; Roy et al., 2020). In the IRLC legumes, the NCR peptides, while in Dalbegioids, the convergently evolved NCR-like peptides represent the vast majority of these host effectors (Van de Velde et al., 2010; Czernic et al., 2015; Montiel et al., 2017; Trujillo et al., 2019).

The *NCR* genes are expressed in the symbiotic nodule cells but in different subsets at sequential stages of the differentiation process (Maunoury et al., 2010; Guefrachi et al., 2014). Immunogold localization and proteome of isolated bacteroids demonstrated undoubtably the presence of NCR peptides in the bacteroids (Van de Velde et al., 2010; Durgo et al., 2015). NCRs are present in all members of the IRLC, but the size and composition of the family vary dramatically among the species from 7 up to ∼700 NCRs (Montiel et al., 2017). In line with the complexity of the NCR family, the morphotype of bacteroids can be swollen, spherical, elongated or both elongated, and branched in different legumes (Montiel et al., 2017).

## The Structure of NCR and NCR-Like Peptides and Their Relatedness to Defensins

**FIGURE 1:**
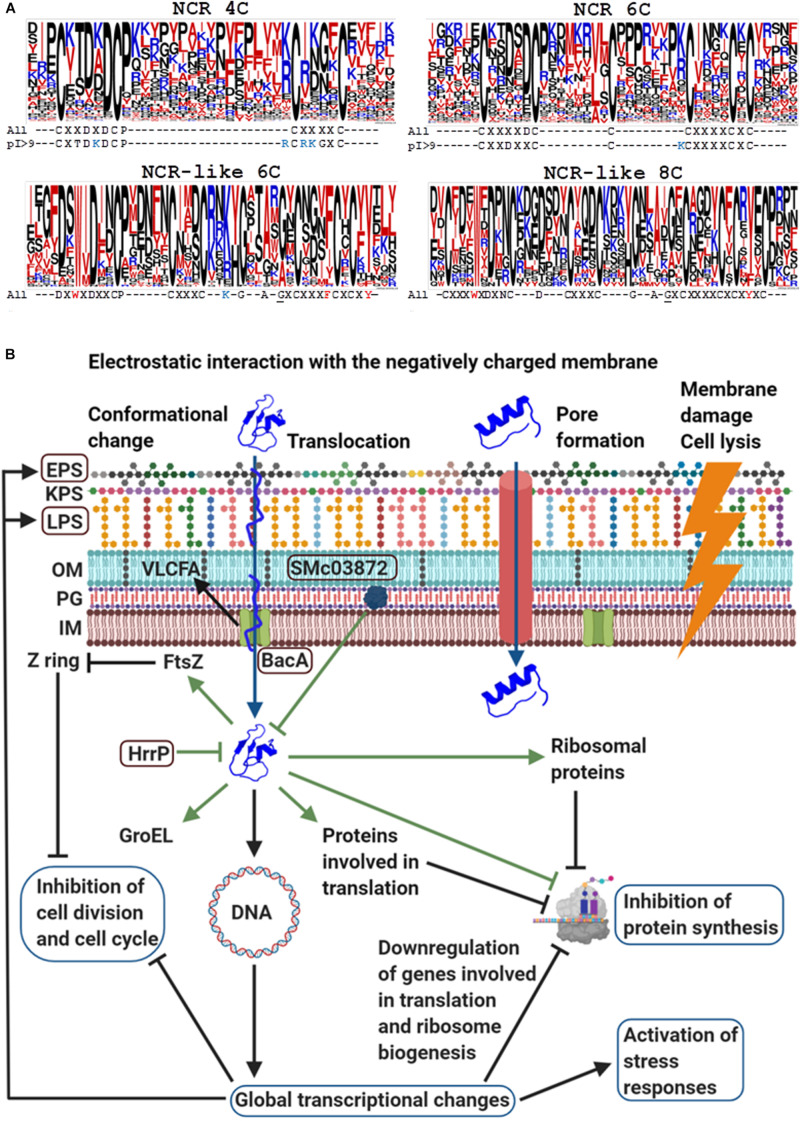
The structure of NCRs, NCR-like peptides **(A)** and the mode of actions of cationic NCRs **(B)**. **(A)** Frequency of amino acids and conserved patterns of cysteines in the mature *M. truncatula* NCRs and NCR-like peptides from Dalbergioid legumes. The height of letters in the stacks indicates the relative frequency of (each amino acid at that position. Color code of amino acids: blue, positively charged (KR) residues; red, hydrophobic (AFILMV) and amphipathic (WY) residues; black, all other amino acids. The underlined G residue in the NCR-like peptides marks the beginning of the γ-core motif. **(B)** The mode of actions of cationic NCRs based on the example of NCR247 (Created with BioRender.com). NCRs can interact with the bacterial membranes and enter the cytosol with or without pore formation or cause membrane damages and cell lysis. Intracellularly NCRs provoke global transcriptional changes and interact with numerous bacterial proteins that collectively affect essential cellular functions. The framed proteins BacA, HrrP, SMc03872, and polysaccharides EPS and LPS protect the symbiotic bacterium partner from the killing action of NCRs.)

There are ∼700 *NCR* genes in the model legume *Medicago truncatula.* The *NCR* genes are usually composed of two exons; the first one codes for a relatively conserved signal peptide while the second one for a highly diverse mature peptide, which contains four or six cysteine residues in conserved positions (Alunni et al., 2007). In 95% of the NCRs, the length of the mature peptides varies between 24 and 65 amino acids but it is mostly 35–50 amino acid long in the majority of NCRs. Figure 1A shows graphical representation of amino acids in multiple alignment of the mature NCR and NCR-like sequences with Jalview version 2.11.0 (Waterhouse et al., 2009) and Clustal X version 2.1 (Larkin et al., 2007) where the height of letters indicates the relative frequency of amino acids at each position (Crooks et al., 2004). Beside the cysteines, only a few amino acids are present in >60% of NCRs, such as the aspartic acid (D) in front of the second cysteine (C_2_) and between C_1_ and C_2_, or proline (P) after C_2_. Due to the high diversity of amino acid composition, the isoelectric points (pI) of the *M. truncatula* NCR peptides vary between 3.5 and 11.25. In *M. truncatula*, 35% of the NCRs are anionic, 23% neutral and 42% cationic and almost equal numbers of genes code for NCRs with four and six cysteines. The high sequence variation also applies to these subgroups. As illustrated for the cationic (pI > 9) NCRs, the presence of the positively charged amino acids (K/R) is characteristic before C_2_ and in front of C_3_ and C_4_ in NCR 4Cs and 6Cs, respectively. Moreover, threonine (T) is frequent after C_1_X in NCR 4Cs but not in the 6Cs.

The cysteines are essential for the symbiotic, *in planta* functions as replacement of a single cysteine with serine resulted in the inactivation of the *Medicago*-specific NCR169 peptide (Horváth et al., 2015). Formation of disulfide bridges between the conserved cysteines could be important structural and functional elements of the NCR peptides. The disulfide bridges can be formed in the endoplasmic reticulum (ER) where enzymes controlling the oxidation of cysteines into disulfide bonds, such as the protein disulfide isomerase and ER oxidoreductin 1, are strongly upregulated (Mergaert et al., 2003; Roux et al., 2014). On the other hand, the symbiotic cells also produce symbiosis-specific thioredoxins that are co-targeted with the NCRs to the cytosol of bacteroids and can reduce the disulfide bonds of NCR peptides (Ribeiro et al., 2017). These observations suggest that NCRs are oxidized in the ER but are reduced within the bacteroids at least partially (Alloing et al., 2018). Accordingly, the redox state of the NCR peptides could represent a further level of complexity in regulating their activites.

The role of cysteines and disulfide bridges was primarily studied in the smallest, 24 amino acid long NCR247 using chemically synthetized peptides and the symbiotic bacterium partner *Sinorhizobium meliloti* in various bioassays. Exchanging the four cysteines for serines (NSR247), altering the position of the disulfide bridges, breaking the bridges by reduction (NCR247_red_) or omitting the cysteines, all affected but to a different extent the peptides’ activities and stability (Haag et al., 2012; Shabab et al., 2016). The disulfide bonds in NCR044 produced in the yeast *Pichia pastoris* were confirmed between C1–C4 and C2–C3, while the three dimensional structure of this peptide was found to be largely dynamic and disordered (Velivelli et al., 2020).

The NCR-like peptides in Dalbergioid legumes, like *Aeschynomene afraspera* and *Aeschynomene indica* are distinct from the IRLC NCRs but play similar roles in provoking terminal differentiation of bacteroids (Czernic et al., 2015). The mature NCR-like peptides are ∼50 amino acid long and have six or eight conserved cysteines and a tryptophan (W) (Figure 1A). These sequences are less divergent and several amino acids are present at >60% frequency at given positions. The NCR-like peptides are anionic or neutral except for two mildly cationic ones.

NCRs and NCR-like peptides resemble defensins, the largest group of plant innate immunity effectors (Sathoff and Samac, 2018). Defensins are also secreted peptides with a length of approximately 45–54 amino acids and 8 or 10 conserved cysteines forming disulfide bonds (Parisi et al., 2019). In spite of variations in the primary sequence, the 3D structure of defensins is conserved. Plant defensins have a γ-core motif (GXCX_3__–__9_C) that is a hallmark related to their antimicrobial properties (Yount and Yeaman, 2004). Interestingly, the γ-core motif is also present in the majority of NCR-like peptides (Figure 1A).

Both the *NCR* and *NCR-like* genes might have originated from an ancestral defensin type gene by gene duplications and fast diversification. The NCR gene family evolution is probably driven by a continuous adaptation to diversifying rhizobium symbionts. In *M. truncatula*, *NCR* genes are present on all chromosomes, and beside long distance duplications, local duplications form small clusters of *NCR* genes. Since many *NCRs* are in the vicinity of transposable elements, transposons might have been involved in the multiplication of *NCR* genes (Satgé et al., 2016).

## Symbiotic Roles of *M. truncatula* NCR Peptides

In the very young symbiotic nodule cells where the endosymbionts multiply, only a few non-cationic *NCR* genes are expressed. When the endosymbiont population reaches a certain density, the endosymbionts enter the differentiation process starting with cell division arrest and cell enlargement (Kondorosi et al., 2013). Changes also occur in the cell envelope and the increased membrane permeability can facilitate the exchange of metabolites between the plant and bacterium. If the differentiation process is incomplete, there is no nitrogen fixation.

One of the major tasks of the NCR peptides is to inhibit and permanently abolish the bacterial cell division. Treatment of *S. meliloti* cultures *in vitro* with synthetic NCRs revealed that cationic peptides like NCR035, NCR055, or NCR247 provoke increased membrane permeability, cell elongation, DNA amplification, and kill ultimately the bacteria (Van de Velde et al., 2010). The mode of action of NCR247 is the best studied one (Figure 1B). Its activation in the nodules coincides with the start of bacteroid differentiation; with cell division arrest and elongation of bacteroids (Farkas et al., 2014). Treatment of *S. meliloti* cultures with 5 μM NCR247 damaged the integrity of bacterial membranes and led to cell death (Farkas et al., 2014; Mikuláss et al., 2016). Cysteines also contribute to the antimicrobial activity of NCR247 and the reduced form is the most effective (Haag et al., 2012; Shabab et al., 2016). NCR247 as well as other cationic NCR peptides provoke formation of outer membrane vesicles (Montiel et al., 2017) but NCR247 at sublethal 1.5 μM concentration, enters the cytosol without pore formation (Farkas et al., 2014).

Treatment of log phase *S. meliloti* cultures or synchonized cells with sublethal concentrations of the reduced and the oxidized forms of NCR247 provoked global transcriptional changes affecting 14–15% of the protein coding sequences (Tiricz et al., 2013; Penterman et al., 2014). Besides general stress response activation, nearly half of the cell cycle genes were affected including critical regulators, such as *dnaA*, *gcrA*, *ctrA* and those involved in septum formation and cell division. Genes involved in translation and particularly in ribosome biogenesis were downregulated. Expression of genes involved in transcriptional regulation, membrane modifications and transport were perturbed.

The Boman index (indicating the protein binding potential) of NCR247 is one of the highest among all known proteins and indeed it possesses extreme protein binding ability (Farkas et al., 2014). Half of the ribosomal proteins and numerous proteins involved in different stages of translation were present in the NCR247 complexes leading to the inhibition of protein synthesis. Its interaction with FtsZ prevented the Z-ring formation and thereby septum assembly and bacterial cell division. Interestingly NCR035, another cationic NCR peptide coexpressed with NCR247, binds to the septum, suggesting that the host plant employs multiple peptides to interfere with specific biological processes, such as the bacterial cell division. NCR247 interacts also with the GroEL chaperone, which is essential for the differentiation of symbiotic cells though it is unknown how the binding of NCR247 affects GroEL functions. Treatment of *S. meliloti* cultures with the most cationic peptide, NCR335, resulted, similarly, in rapid downregulation of genes involved in basic cellular functions, such as transcription-translation and energy production, as well as upregulation of genes involved in stress and oxidative stress responses and membrane transport (Tiricz et al., 2013).

While cationic NCRs exhibited toxicity *in vitro* for rhizobia, none of the tested anionic peptides, except for NCR211 affected the survival of rhizobia (Kim et al., 2015). At present it is unkown how NCR211 and the non-cationic NCR-like peptides exert antimicrobial properties.

In the nodule cells, the rhizobia are viable and are likely to be exposed to lower concentrations of NCRs than those used in the *in vitro* assays. Moreover, the bacteria have evolved various mechanisms against the toxicity of NCRs. BacA is essential for the survival of bacteroids in *M. truncatula* (Haag et al., 2011). BacA is an ABC transporter protein, which promotes uptake and translocation of NCRs from the membrane to the cytosol that might diminish the membrane damage and keep the bacteria alive (Guefrachi et al., 2015; Barrière et al., 2017). Components of the cell envelope also provide protection, such as lipopolysaccharides (LPS) together with the BacA mediated synthesis of very long chain fatty acids (VLCFA), high molecular weight succinoglycans in the exopolysaccharide (EPS) layer and other membrane constituents (Arnold et al., 2017, 2018; Montiel et al., 2017). Proteolytic degradation of NCRs by the bacterial HrrP and SMc03872 represents another level of resistance (Price et al., 2015; Arnold et al., 2017) though the oxidized forms are more stable (Shabab et al., 2016).

## Antibacterial Spectrum of NCRs

Cationic NCRs are in many respects similar to membrane-permeabilizing cationic antimicrobial peptides whose net charge ranges from +2 to +9 and facilitaties their interaction with the negatively charged bacterial membranes. Most antibacterial tests have been carried out with NCR247 (net charge +6) and NCR335 (net charge +14) which were classified with four and two different AMP prediction tools as AMPs, respectively (Farkas et al., 2017). NCR335 is unusual because it is 64 amino acid long and only its C-terminal half carries the conserved cysteine pattern of NCRs. The antimicrobial activity of chemically synthetized NCRs has been tested against a broad panel of Gram-negative (*Escherichia coli*, *Salmonella enterica*, *Pseudomonas aeruginosa*, *Pseudomonas syringae* pv. *tomato*, *Xanthomonas campestris*, *Agrobacterium tumefaciens*, *Chlamydia trachomatis*) and Gram-positive (*Bacillus megaterium*, *Bacillus cereus*, *Bacillus subtilis*, *Listeria monocytogenes*, *Staphylococcus aureus*, *Clavibacter michiganensis*) bacteria, including diverse human/animal and plant pathogens. The peptides, added to 10^7^, bacteria for 3 h, killed to various extent all these tested bacteria resulting in their complete elimination or decrease in the number of surviving cells from one to several orders of magnitude, depending on the strain and the peptide (Tiricz et al., 2013; Balogh et al., 2014). In general, cationic NCR peptides with pI >9.0 seem to have antibacterial activities, however, their antimicrobial spectrum was only partially overlapping indicating that in addition to their positive charge, their amino acid composition and primary sequence also contribute to the strength and spectrum of antibacterial activities. Due to the multiple bacterial targets of NCRs, there is little chance for development of resistance against them.

## Antibacterial Potential of NCR247-Based Chimeric Peptides Is Comparable to Third Generation Antibiotics

Skin and soft tissue infections are mainly caused by ESKAPE bacteria which are resistant to most antibiotics (Pfalzgraff et al., 2018). Based on the different mode of action and broad spectrum of NCRs it is conceivable that they may also be able to kill these resistant pathogens. The antibacterial activity of chemically synthesized NCR247 and NCR247-derivatives was investigated against ESKAPE strains (*Enterococcus faecalis*, *S. aureus*, *Klebsiella pneumoniae*, *Acinetobacter baumannii*, *P. aeruginosa*) and *E. coli, L. monocytogenes*, and *S. enterica* (Jenei et al., 2020; Table 1). The minimal bactericidal concentration of NCR247 was 3.1 μM against *P*. *aeruginosa* and 6.3 μM against *S. aureus* and *E. coli* while killing of the other bacteria required higher concentrations. The C-terminal half of NCR247 (NCR247C) retained its activity on *E. coli* but lost its effectiveness on other bacteria. To improve its antimicrobial properties, NCR247C was fused with NCR335_7__–__19_ (X1) or mastoparan_4__–__14_ (X2) deriving from the 14 amino acid long mastoparan, a membranolytic peptide toxin from wasp venom. Each of these chimeric peptides possessed higher antibacterial efficacy and affected the antimicrobial spectrum. In the case of X1-NCR247C the minimal bactericidal concentrations (MBC) varied between 1.6 and 12.5 μM. C- or N-terminal fusion of NCR247C with X2 made the chimeric peptides very effective on most strains at 1.6 and 3.1 μM MBCs. The MBCs of these chimeric derivatives were much lower than that of the classical antibiotic carbenicillin, and were comparable or even more effective than levofloxacin, a third generation antibiotic (Jenei et al., 2020).

**TABLE 1:** The amino acid sequence of NCR247 and its derivatives (A) and the minimal bactericidal concentrations (MBCs) of these peptides and antibiotics (in μM) on different pathogens (B) (from Jenei et al., 2020).

**(A)**	**Name**			**Amino acid sequence**				
	NCR247			RNGCIVDPRCPY**QQCRRPLYCRRR**				
	**NCR247C**			**QQCRRPLYCRRR**				
	**X1** (NCR335_7__–__19_)-**NCR247C**			RPLNFKMLRFWGQ**QQCRRPLYCRRR**				
	**NCR247C**-**X2** (Mastoparan_4__–__14_)			**QQCRRPLYCRRR**KALAALAKKIL				
	**X2** (Mastoparan_4__–__14_)-**NCR247C**			KALAALAKKIL**QQCRRPLYCRRR**				
	**X2** (Mastoparan_4__–__14_)			KALAALAKKIL				

**(B)**								

**Peptides/Antibiotics**	***E. f.***	***S. a.***	***K. p.***	***A. b.***	***P. a.***	***E. c.***	***L. m.***	***S. e.***

NCR247	>25	**6.3**	>25	12.5	**3.1**	**6.3**	>25	25
NCR247C	>25	> 25	>25	25	25	**6.3**	>25	> 25
**X1-NCR247C**	**6.3**	**3.1**	12.5	**3.1**	**3.1**	**3.1**	**3.1**	**1.6**
**NCR247C-X2**	25	**3.1**	**6.3**	**3.1**	**3.1**	**1.6**	**1.6**	**1.6**
**X2-NCR247C**	**3.1**	**3.1**	**6.3**	**3.1**	**3.1**	**3.1**	**3.1**	**3.1**
**X2**	>25	25	>25	25	**6.3**	>25	25	>25
**Cb**	5120	640	>10,240	5120	10240	1280	80	640
**Lvx**	160	**2.5**	320	20	**1.3**	**5.0**	320	**1.3**

**E. f., Enterococcus faecalis; S. a., Staphylococcus aureus; K. p., Klebsiella pneumoniae; A. b., Acinetobacter baumannii; P. a., Pseudomonas aeruginosa; E. c., Escherichia coli; L. m., Listeria monocytogenes; S. e., Salmonella enterica. Cb, carbenicillin; Lvx, levofloxacin. MBCs below 10 μM are in bold.**

The killing activity of the NCR247-based chimeric peptides occurred within 0.1–5 min. While the antimicrobial activity of cationic peptides is generally attenuated by the presence of divalent cations and higher salt concentrations (Hancock and Sahl, 2006), the bactericidal activity of these chimeric peptides was maintained in Mueller Hinton broth. Importantly, these peptides did not have hemolytic activity or cytotoxicity on human cells (Jenei et al., 2020).

## Antifungal Activity of NCRs

The relatedness of NCRs to antimicrobial peptides, particularly to plant defensins protecting the plants mostly against fungal infections suggests that NCRs also have antifungal activity. Among 19 NCR peptides with pI ranging from 3.61 to 11.22, nine with pI >9.5 inhibited the growth and the survival of both the yeast and filamentous forms of *Candida albicans*, one of the most common opportunistic human pathogens (Ördögh et al., 2014). The minimal fungicidal concentrations of the most effective peptides (NCR335, NCR044) were between 1 and 3 μM. Treatment of *C. albicans*-infected vaginal epithelial cells with NCR335, NCR247, or NCR192 for 3 h prevented epithelial cell death induced by *C. albicans*. The concentrations required for killing the fungus did not affect survival of human cells. The anticandidal activity of NCR peptides was achieved by permeabilization of the fungal membrane and interactions with multiple intracellular targets. Cationic NCR peptides were also active on *Aspergillus niger, Candida crusei, Candida parapsilosis, Fusarium graminearum*, *Rhizopus stolonifer var. stolonifer*, however, their antifungal spectrum and efficacity varied indicating that, similarly, to the bactericidal action, in addition to the pI, the amino acid sequence also contributes to the antifungal properties (Kondorosi-Kuzsel et al., 2010). NCR044 exhibited strong fungicidal activity against the plant pathogen *Botrytis cinerea* and several *Fusarium* species (Velivelli et al., 2020). The inhibitory concentration of NCR044 varied between 0.52 and 1.93 μM. NCR044 interacts with the *B. cinerea* cell wall and the membrane phospholipids, then it translocates to the cytoplasm and localizes to the nucleolus. It provokes production of reactive oxygen species and might interfere with protein synthesis. Thus, both the antibacterial and the antifungal activities of NCRs rely on multistep actions. In lettuce leaves and rose petal assays, NCR044 provided resistance to *B. cinerea*. These findings together with the economical production of NCR044 in *P. pastoris* paves the way to use NCRs in agriculture for plant protection (Velivelli et al., 2020).

## Conclusion

Antimicrobial resistance is a global healthcare threat. Many people die from incurable infections and with the lack of appropriate antibiotics we might return to the pre-antibiotic era. AMPs represent a new hope with their rapid killing and broad spectrum activity against multidrug resistant (MDR) pathogens. AMPs, like the cationic NCRs, are multifunctional. They can interact with the membranes with or without membrane permeabilization and intracellularly they can affect transcription, translation, enzyme activities causing ultimately microbial death (Mwangi et al., 2019). A few AMPs with potent activity against MDR species are in clinical use like colistin, one of the last-resort drugs (Pfalzgraff et al., 2018; Mwangi et al., 2019). Toxicity of AMPs is, however, a major drawback and many AMPs are limited to topical application. To the 3011 AMPs in the antimicrobial peptide database (Wang, 2020), legumes can add several ten thousands of natural AMPs produced in the symbiotic cells. Legumes are mostly edible plants and NCRs are apparently not toxic for human cells while many of them kill pathogenic bacteria and fungi very effectively with multi-target actions. In laboratory conditions, NCRs or their derivatives, such as various chimeric peptides have similar or even superior antimicrobial properties than third generation antibiotics. Exploring their potential might help to fight against existing and unforeseen bacterial, fungal and possibly viral infections both in medicine and agriculture.

## Author Contributions

ÉK conceptualized the manuscript. RL and SK analyzed the peptide sequences and provided the Figure 1. All authors contributed to the manuscript.

## Conflict of Interest

The authors declare that the research was conducted in the absence of any commercial or financial relationships that could be construed as a potential conflict of interest.

## References

[B1] Alloing G. Mandon K. Boncompagni E. Montrichard F. Frendo P. (2018). Involvement of glutaredoxin and thioredoxin systems in the nitrogen-fixing symbiosis between legumes and rhizobia. *Antioxidants* 7:182. 10.3390/antiox7120182 30563061 PMC6315971

[B2] Alunni B. Kevei Z. Redondo-Nieto M. Kondorosi A. Mergaert P. Kondorosi E. (2007). Genomic organization and evolutionary insights on GRP and NCR genes, two large nodule-specific gene families in *Medicago truncatula*. *Mol. Plant. Microbe Interact.* 20 1138–1148. 10.1094/MPMI-20-9-1138 17849716

[B3] Arnold M. F. F. Penterman J. Shabab M. Chen E. J. Walker G. C. (2018). Important late-stage symbiotic role of the *Sinorhizobium meliloti* exopolysaccharide succinoglycan. *J. Bacteriol.* 200:e00665-17. 10.1128/JB.00665-17 29632097 PMC5996692

[B4] Arnold M. F. F. Shabab M. Penterman J. Boehme K. L. Griffitts J. S. Walker G. C. (2017). Genome-wide sensitivity analysis of the microsymbiont Sinorhizobium meliloti to symbiotically important, defensin-like host peptides. *mBio* 8:e01060-17. 10.1128/mBio.01060-17 28765224 PMC5539429

[B5] Balogh E. Mosolygó T. Tiricz H. Szabó Á Karai A. Kerekes F. (2014). Anti-chlamydial effect of plant peptides. *Acta Microbiol. Immunol. Hung.* 61 229–239. 10.1556/AMicr.61.2014.2.12 24939689

[B6] Barrière Q. Guefrachi I. Gully D. Lamouche F. Pierre O. Fardoux J. (2017). Integrated roles of BclA and DD-carboxypeptidase 1 in *Bradyrhizobium* differentiation within NCR-producing and NCR-lacking Root Nodules. *Sci. Rep.* 7:9063. 10.1038/s41598-017-08830-0 28831061 PMC5567381

[B7] Crooks G. E. Hon G. Chandonia J.-M. Brenner S. E. (2004). WebLogo: a sequence logo generator. *Genome Res.* 14 1188–1190. 10.1101/gr.849004 15173120 PMC419797

[B8] Czernic P. Gully D. Cartieaux F. Moulin L. Guefrachi I. Patrel D. (2015). Convergent evolution of endosymbiont differentiation in Dalbergioid and Inverted Repeat-Lacking Clade legumes mediated by nodule-specific cysteine-rich peptides. *Plant Physiol.* 169 1254–1265. 10.1104/pp.15.00584 26286718 PMC4587450

[B9] Durgo H. Klement E. Hunyadi-Gulyas E. Szucs A. Kereszt A. Medzihradszky K. F. (2015). Identification of nodule-specific cysteine-rich plant peptides in endosymbiotic bacteria. *Proteomics* 15 2291–2295. 10.1002/pmic.201400385 25690539

[B10] Farkas A. Maróti G. Dürgõ H. Györgypál Z. Lima R. M. Medzihradszky K. F. (2014). *Medicago truncatula* symbiotic peptide NCR247 contributes to bacteroid differentiation through multiple mechanisms. *Proc. Natl. Acad. Sci. U.S.A.* 111 5183–5188. 10.1073/pnas.1404169111 24706863 PMC3986156

[B11] Farkas A. Maróti G. Kereszt A. Kondorosi É (2017). Comparative analysis of the bacterial membrane disruption effect of two natural plant antimicrobial peptides. *Front. Microbiol.* 8:51. 10.3389/fmicb.2017.00051 28167938 PMC5253368

[B12] Graham P. H. Vance C. P. (2003). Legumes: importance and constraints to greater use. *Plant Physiol.* 131 872–877. 10.1104/pp.017004 12644639 PMC1540286

[B13] Guefrachi I. Nagymihaly M. Pislariu C. I. Van de Velde W. Ratet P. Mars M. (2014). Extreme specificity of NCR gene expression in *Medicago truncatula*. *BMC Genomics* 15:712. 10.1186/1471-2164-15-712 25156206 PMC4168050

[B14] Guefrachi J. Pierre O. Timchenko T. Alunni B. Barrière Q. Czernic P. (2015). *Bradyrhizobium* BclA is a peptide transporter required for bacterial differentiation in symbiosis with *Aeschynomene* Legumes. *Mol. Plant Microbe. Interact.* 28, 1155–1166. 10.1094/MPMI-04-15-0094-R 26106901

[B15] Haag A. F. Baloban M. Sani M. Kerscher B. Pierre O. Farkas A. (2011). Protection of *Sinorhizobium* against host cysteine-rich antimicrobial peptides is critical for symbiosis. *PLoS Biol.* 9:e1001169. 10.1371/journal.pbio.1001169 21990963 PMC3186793

[B16] Haag A. F. Kerscher B. Dall’Angelo S. Sani M. Longhi R. Baloban M. (2012). Role of cysteine residues and disulfide bonds in the activity of a legume root nodule-specific, cysteine-rich peptide. *J. Biol. Chem.* 287 10791–10798. 10.1074/jbc.M111.311316 22351783 PMC3322895

[B17] Hancock R. E. W. Sahl H.-G. (2006). Antimicrobial and host-defense peptides as new anti-infective therapeutic strategies. *Nat. Biotechnol.* 24 1551–1557. 10.1038/nbt1267 17160061

[B18] Horváth B. Domonkos Á Kereszt A. Szûcs A. Ábrahám E. Ayaydin F. (2015). Loss of the nodule-specific cysteine rich peptide, NCR169, abolishes symbiotic nitrogen fixation in the *Medicago truncatula* dnf7 mutant. *Proc. Natl. Acad. Sci. U.S.A.* 112 15232–15237. 10.1073/pnas.1500777112 26401023 PMC4679056

[B19] Jenei S. Tiricz H. Szolomájer J. Tímár E. Klement É Al Bouni M. A. (2020). Potent chimeric antimicrobial derivatives of the *Medicago truncatula* NCR247 symbiotic peptide. *Front. Microbiol.* 11:270. 10.3389/fmicb.2020.00270 32153547 PMC7047876

[B20] Kim M. Chen Y. Xi J. Waters C. Chen R. Wang D. (2015). An antimicrobial peptide essential for bacterial survival in the nitrogen-fixing symbiosis. *Proc. Natl. Acad. Sci. U.S.A.* 112 15238–15243. 10.1073/pnas.1500123112 26598690 PMC4679048

[B21] Kondorosi E. Mergaert P. Kereszt A. (2013). A paradigm for endosymbiotic life: cell differentiation of *Rhizobium* bacteria provoked by host plant factors. *Annu. Rev. Microbiol.* 67 611–628. 10.1146/annurev-micro-092412-155630 24024639

[B22] Kondorosi-Kuzsel E. Mergaert P. Van de Velde W. Maroti G. Farkas A. Kereszt A. (2010). *Nodule specific medicago peptides having antimicrobial activity and pharmaceutical compositions containing the same. International Patent EP2442823B1, WO2010146067Al.*

[B23] Larkin M. A. Blackshields G. Brown N. P. Chenna R. McGettigan P. A. McWilliam H. (2007). Clustal W and Clustal X version 2.0. *Bioinformatics* 23 2947–2948. 10.1093/bioinformatics/btm404 17846036

[B24] Maunoury N. Redondo-Nieto M. Bourcy M. Van de Velde W. Alunni B. Laporte P. (2010). Differentiation of symbiotic cells and endosymbionts in *Medicago truncatula* nodulation are coupled to two transcriptome-switches. *PLoS One* 5:e9519. 10.1371/journal.pone.0009519 20209049 PMC2832008

[B25] Mergaert P. (2018). Role of antimicrobial peptides in controlling symbiotic bacterial populations. *Nat. Prod. Rep.* 35 336–356. 10.1039/C7NP00056A 29393944

[B26] Mergaert P. Nikovics K. Kelemen Z. Maunoury N. Vaubert D. Kondorosi A. (2003). A novel family in *Medicago truncatula* consisting of more than 300 nodule-specific genes coding for small, secreted polypeptides with conserved cysteine motifs. *Plant Physiol.* 132 161–173. 10.1104/pp.102.018192 12746522 PMC166962

[B27] Mergaert P. Uchiumi T. Alunni B. Evanno G. Cheron A. Catrice O. (2006). Eukaryotic control on bacterial cell cycle and differentiation in the Rhizobium–legume symbiosis. *Proc. Natl. Acad. Sci. U.S.A.* 103 5230–5235. 10.1073/pnas.0600912103 16547129 PMC1458823

[B28] Mikuláss K. R. Nagy K. Bogos B. Szegletes Z. Kovács E. Farkas A. (2016). Antimicrobial nodule-specific cysteine-rich peptides disturb the integrity of bacterial outer and inner membranes and cause loss of membrane potential. *Ann. Clin. Microbiol. Antimicrob.* 15:43. 10.1186/s12941-016-0159-8 27465344 PMC4964015

[B29] Montiel J. Downie J. A. Farkas A. Bihari P. Herczeg R. Bálint B. (2017). Morphotype of bacteroids in different legumes correlates with the number and type of symbiotic NCR peptides. *Proc. Natl. Acad. Sci. U.S.A.* 114 5041–5046. 10.1073/pnas.1704217114 28438996 PMC5441718

[B30] Mwangi J. Hao X. Lai R. Zhang Z.-Y. (2019). Antimicrobial peptides: new hope in the war against multidrug resistance. *Zool. Res.* 40 488–505. 10.24272/j.issn.2095-8137.2019.062 31592585 PMC6822926

[B31] Oono R. Denison R. F. (2010). Comparing symbiotic efficiency between swollenversus nonswollen rhizobial bacteroids. *Plant Physiol.* 154 1541–1548. 10.1104/pp.110.163436 20837702 PMC2971627

[B32] Ördögh L. Vörös A. Nagy I. Kondorosi É Kereszt A. (2014). Symbiotic plant peptides eliminate Candida albicans both in vitro and in an epithelial infection model and inhibit the proliferation of immortalized human cells. *BioMed Res. Int.* 2014:320796. 10.1155/2014/320796 25243129 PMC4163382

[B33] Parisi K. Shafee T. M. A. Quimbar P. van der Weerden N. L. Bleackley M. R. Anderson M. A. (2019). The evolution, function and mechanisms of action for plant defensins. *Semin. Cell Dev. Biol.* 88 107–118. 10.1016/j.semcdb.2018.02.004 29432955

[B34] Penterman J. Abo R. P. De Nisco N. J. Arnold M. F. F. Longhi R. Zanda M. (2014). Host plant peptides elicit a transcriptional response to control the *Sinorhizobium meliloti* cell cycle during symbiosis. *Proc. Natl. Acad. Sci. U.S.A.* 111 3561–3566. 10.1073/pnas.1400450111 24501120 PMC3948309

[B35] Pfalzgraff A. Brandenburg K. Weindl G. (2018). Antimicrobial peptides and their therapeutic potential for bacterial skin infections and wounds. *Front. Pharmacol.* 9:281. 10.3389/fphar.2018.00281 29643807 PMC5882822

[B36] Price P. A. Tanner H. R. Dillon B. A. Shabab M. Walker G. C. Griffitts J. S. (2015). Rhizobial peptidase HrrP cleaves host-encoded signaling peptides and mediates symbiotic compatibility. *Proc. Natl. Acad. Sci. U.S.A.* 112 15244–15249. 10.1073/pnas.1417797112 26401024 PMC4679054

[B37] Ribeiro C. W. Baldacci-Cresp F. Pierre O. Larousse M. Benyamina S. Lambert A. (2017). Regulation of differentiation of nitrogen-fixing bacteria by microsymbiont targeting of plant thioredoxin s1. *Curr. Biol.* 27 250–256. 10.1016/j.cub.2016.11.013 28017611

[B38] Roux B. Rodde N. Jardinaud M.-F. Timmers T. Sauviac L. Cottret L. (2014). An integrated analysis of plant and bacterial gene expression in symbiotic root nodules using laser-capture microdissection coupled to RNA sequencing. *Plant J.* 77 817–837. 10.1111/tpj.12442 24483147

[B39] Roy P. Achom M. Wilkinson H. Lagunas B. Gifford M. L. (2020). Symbiotic outcome modified by the diversification from 7 to over 700 nodule-specific cysteine-rich peptides. *Genes* 11:348. 10.3390/genes11040348 32218172 PMC7230169

[B40] Satgé C. Moreau S. Sallet E. Lefort G. Auriac M.-C. Remblière C. (2016). Reprogramming of DNA methylation is critical for nodule development in *Medicago truncatula*. *Nat. Plants* 2 1–10. 10.1038/nplants.2016.166 27797357

[B41] Sathoff A. E. Samac D. A. (2018). Antibacterial activity of plant defensins. *Mol. Plant. Microbe. Interact.* 32 507–514. 10.1094/MPMI-08-18-0229-CR 30501455

[B42] Shabab M. Arnold M. F. F. Penterman J. Wommack A. J. Bocker H. T. Price P. A. (2016). Disulfide cross-linking influences symbiotic activities of nodule peptide NCR247. *Proc. Natl. Acad. Sci. U.S.A.* 113 10157–10162. 10.1073/pnas.1610724113 27551097 PMC5018749

[B43] Tiricz H. Szûcs A. Farkas A. Pap B. Lima R. M. Maróti G. (2013). Antimicrobial nodule-specific cysteine-rich peptides induce membrane depolarization-associated changes in the transcriptome of *Sinorhizobium meliloti*. *Appl. Environ. Microbiol.* 79 6737–6746. 10.1128/AEM.01791-13 23995935 PMC3811505

[B44] Trujillo D. I. Silverstein K. A. T. Young N. D. (2019). Nodule-specific PLAT domain proteins are expanded in the *Medicago* lineage and required for nodulation. *New Phytol.* 222 1538–1550. 10.1111/nph.15697 30664233

[B45] Van de Velde W. Zehirov G. Szatmari A. Debreczeny M. Ishihara H. Kevei Z. (2010). Plant peptides govern terminal differentiation of bacteria in symbiosis. *Science* 327 1122–1126. 10.1126/science.1184057 20185722

[B46] Velivelli S. L. S. Czymmek K. J. Li H. Shaw J. B. Buchko G. W. Shah D. M. (2020). Antifungal symbiotic peptide NCR044.1 exhibits unique structure and multi-faceted mechanisms of action that confer plant protection. *bioRxiv [Preprint]* 10.1101/2020.02.19.956318 PMC7354933 32571919

[B47] Wang G. (2020). The antimicrobial peptide database provides a platform for decoding the design principles of naturally occurring antimicrobial peptides. *Protein Sci.* 29 8–18. 10.1002/pro.3702 31361941 PMC6933855

[B48] Waterhouse A. M. Procter J. B. Martin D. M. A. Clamp M. Barton G. J. (2009). Jalview version 2: a multiple sequence alignment editor and analysis workbench. *Bioinformatics* 25 1189–1191. 10.1093/bioinformatics/btp033 19151095 PMC2672624

[B49] Yount N. Y. Yeaman M. R. (2004). Multidimensional signatures in antimicrobial peptides. *Proc. Natl. Acad. Sci. U.S.A.* 101 7363–7368. 10.1073/pnas.0401567101 15118082 PMC409924

